# Heat shock protein 10 as a chaperone modulating α‐synuclein amyloid fibril formation

**DOI:** 10.1002/pro.70452

**Published:** 2026-01-20

**Authors:** Johan N. K. Larsson, Ranjeet Kumar, Fiamma Ayelen Buratti, Sofie Nyström, Pernilla Wittung‐Stafshede, Per Hammarström

**Affiliations:** ^1^ Department of Physics, Chemistry and Biology Linköping University Linköping Sweden; ^2^ Department of Life Sciences Chalmers University of Technology Gothenburg Sweden; ^3^ Chemistry Department Rice University Houston Texas USA; ^4^ SciLifeLab Linköping University Linköping Sweden

**Keywords:** alpha‐synuclein, amyloid fibril, atomic force microscopy, heat shock protein, molecular chaperone, small angle X‐ray scattering

## Abstract

HSP10 is a well‐known human co‐chaperone that interacts with HSP60 to comprise the HSP60/10 chaperonin complex which upholds mitochondrial proteostasis. HSP10 also demonstrates independent roles in binding to misfolded proteins and interacts with several amyloidogenic client proteins. Using a variety of biophysical and biochemical methods, we studied the interactions of HSP10 with the amyloidogenic protein α‐synuclein (α‐syn) associated with Parkinson's disease. HSP10 efficiently inhibited fibril formation of wild type (WT) and disease‐mutant A30P α‐syn at sufficient concentrations of chaperone by both binding to α‐syn monomers and by blocking secondary nucleation on fibril surfaces. However, under sub‐stoichiometric conditions, below 1:5 (HSP10:α‐syn), the chaperone sequestered multiple A30P α‐syn monomers and thereby promoted nucleation of fibril formation with a magnitude comparable to the efficacy of seeding with preformed fibrils. The fibril formation acceleration effect of the HSP10 chaperone was client‐specific as it was observed for A30P but not WT α‐syn. Our results broaden the scope of HSP10 chaperone activity and can have implications for disease onset in synucleinopathies.

## 1 INTRODUCTION

Amyloidogenic aggregation of the protein α‐synuclein (α‐syn) is a key hallmark in many neurodegenerative diseases. The most widespread synucleinopathy is Parkinson's disease. Aggregation of α‐syn is also linked to Lewy body dementia, multiple system atrophy, and pure autonomic failure (Brás et al., 2020). The neurodegenerative activity of α‐syn aggregates partly stems from intracellular interactions with organelles of the cell such as lysosomes and mitochondria, where the effect on mitochondria and the induction of mitochondrial dysfunction have been well studied and observed for decades (Schapira, 1993). One of the normal cellular responses to combat this issue is to implement molecular chaperones, with the outcome of limiting the accumulation of aggregation and misfolding proteins (Hu et al., 2021). For mitochondria, the main chaperone system upholding the proteostasis is the HSP60/10 chaperonin complex consisting of HSP60 and the homoheptameric co‐chaperone HSP10 (Bie et al., 2020; Singh et al., 2024). HSP10 from the *HSPE1* gene is also known as Heat Shock Protein Family E Member 1, mtHSP10, and CPN10. Homologues of this system can be found in all living organisms with a very high structural conservation between different organisms (Hunt et al., 1996). Furthermore, the amino acid sequence of HSP10 is extremely well conserved and, to that effect, only one individual mutation carrier has been reported where the patient exhibited a neurodevelopmental disorder (Bie et al., 2016). The complete role of HSP10 is not fully understood, although its role as a co‐chaperone to HSP60 and formation of the chaperonin complex is well characterized (Singh et al., 2024). HSP10 has also been found outside the mitochondria and appears heavily involved in regulation of autoimmunity and pregnancy (Morton, 1998).

The HSP60/10 chaperonin is vital in upholding normal mitochondrial function by regulating the proteostasis and folding of hundreds of client proteins (Bie et al., 2020; Singh et al., 2024). Loss‐of‐function of the HSP60/10 chaperonin has earlier been shown to be affected in Parkinson's disease, as a consequence of entrapment of the co‐chaperone HSP10 in α‐syn aggregates (Szegő et al., 2019). Overexpression of HSP10 mitigated pathological effects of α‐syn expression (Szegő et al., 2019). The levels of HSP10 have also been documented to be altered in other amyloid diseases, such as Alzheimer's disease and diabetes type 2 (Hashimoto et al., 2012; Wardelmann et al., 2021). Mitochondria are severely affected by the accumulation of misfolded α‐syn, and the spread of α‐syn aggregates will continuously damage mitochondria. Moreover, the loss of functional HSP10 hinders its function to uphold the proteostasis together with HSP60, leading to an accumulation of reactive oxygen species in the mitochondria. Previously, we have shown that HSP10 can inhibit aggregation of Amyloid beta (Aβ) and prion protein independently of HSP60 (Larsson et al., 2022). The proposed mechanism was that HSP10 acted as a holdase using its mobile loops that are normally used in binding to HSP60 to associate with the amyloid protein. This proposed mechanism of HSP10 binding to protein clients during folding has previously been documented in the context of bacterial HSP10 (Moparthi et al., 2014, 2016) and could be a mechanism that explains how HSP10 independently from HSP60 regulates autoimmunity and its presence in pregnancy (Corrao et al., 2010). We were curious about the generality of the holdase function of HSP10 and the chaperoning effect on different amyloid proteins. This led us to investigate this function with α‐syn. Herein, we performed a systematic biophysical study of human HSP10 interacting with α‐syn using a different experimental setup from previously published data (Szegő et al., 2019). Using WT α‐syn and a familial Parkinsons disease (fPD) associated point mutation A30P α‐syn (Krüger et al., 1998), we discovered rather surprising differences in the chaperone interactions being client mutation dependent, suggesting specific interactions being dictated by conformational parameters.

## 2 RESULTS

### 2.1 HSP10 modulation of α‐synuclein fibril formation rates

**FIGURE 1:**
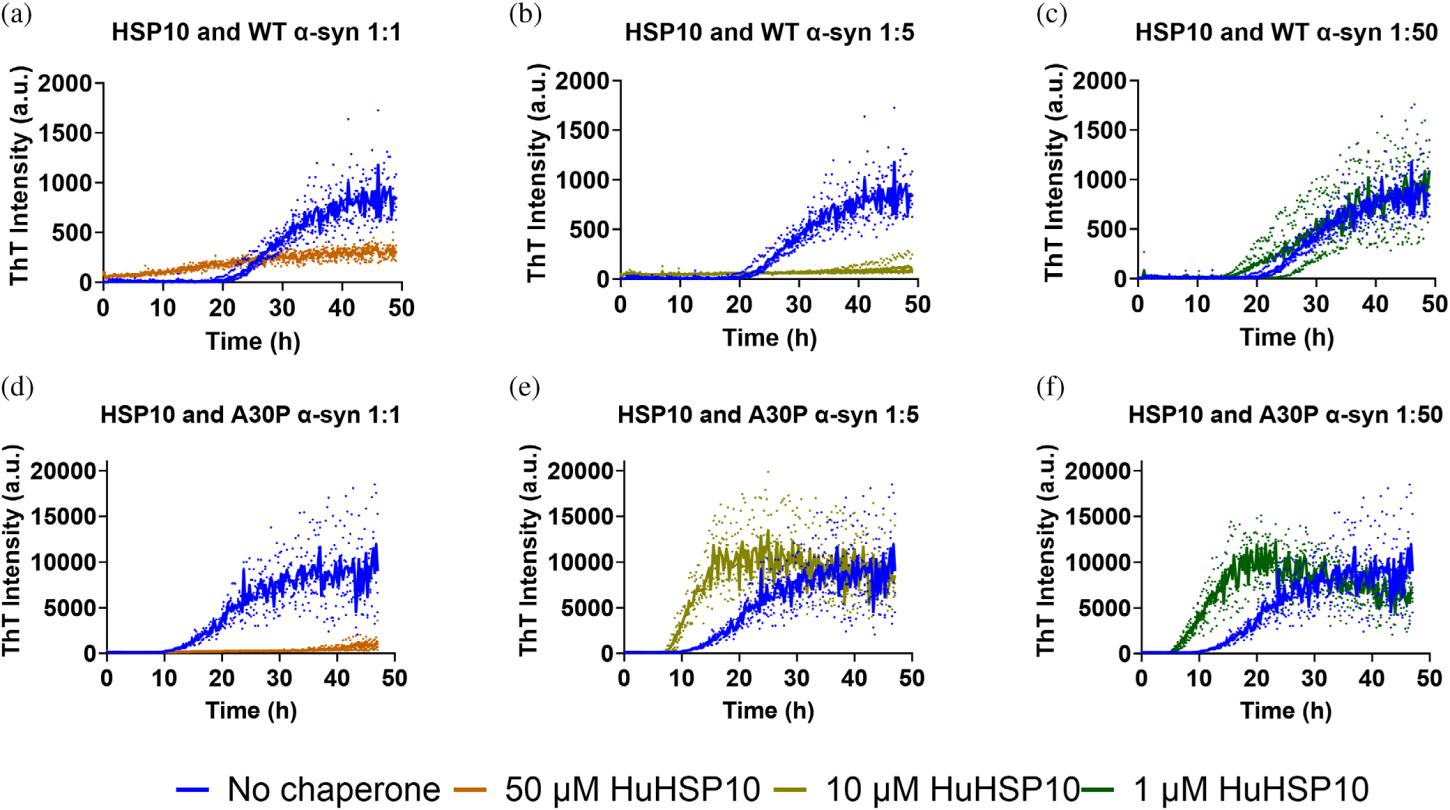
Fibril formation kinetics by ThT fluorescence under different chaperone and α‐syn ratios. Herein, 50 μM WT α‐syn and A30P α‐syn were aggregated in the absence or presence of HSP10 at concentrations of: 50 μM (a) and (d), 10 μM (b), and (f) and 1 μM (c) and (f). Aggregation kinetics without chaperone are shown in the blue traces in every graph. The kinetics was conducted in a half area 96 well plate (corning 3881) at 37°C with shaking in between the measurements. All samples were prepared in four replicates. The linear rise in ThT intensity for the highest HSP10 concentration visible in (a) could be due to HSP10 self‐association at 50 μM.

The fibril formation kinetics of α‐syn in the presence of HSP10 were studied by ThT fluorescence. Reactions were conducted under shaking conditions (37°C, 50 μM) using WT α‐syn and the fPD mutant A30P. We noted that the final ThT intensities were higher for A30P than for WT α‐syn in all experiments. In the absence of chaperone, the halftimes of fibril formation (*T*_1/2_) were 26.8 ± 3.7 h for WT and 24.9 ± 4.6 h for A30P α‐syn taking all reaction replicates into account (*n* = 8) (Table 1). Due to the variation in fibril formation rates, we compared identical samples run on the same plate for WT and A30P α‐syn, respectively, with and without addition of HSP10 chaperone. HSP10 was added at different concentrations to investigate the chaperone modulating effect on the amyloid fibril formation of α‐syn. HSP10 showed complete inhibition (*T*_1/2_ >48 h) of WT α‐syn when investigating the ratios 1:1 and 1:5 (HSP10:α‐syn) (Figure 1a,b and Table 1), but the inhibitory activity was abolished at the 1:50 ratio (Figure 1c). When investigating the aggregation kinetics of A30P α‐syn, the inhibitory range was narrower. HSP10 inhibited fibril formation at a 1:1 ratio (Figure 1d), but the inhibition activity was abolished already at the 1:5 ratio, where surprisingly an accelerated amyloid fibril formation rate was observed (Figure 1e) decreasing the *T*_1/2_ to 11.2 ± 1 h. The accelerated kinetics persisted for HSP10 at the ratio of 1:50 for the A30P α‐syn (Figure 1f). The fibril formation acceleration effects by HSP10 were of the same magnitude as seeding with preformed fibrils (Table 1). Hence, the shift from inhibition to acceleration of fibril formation for A30P α‐syn occurred in a narrow concentration range.

**TABLE 1:** Fibril formation halftimes (*T*_1/2_) in hours (h) for α‐syn by ThT fluorescence.

[HSP10] (μM)	WT α‐syn (*T*_1/2_ h)	A30P α‐syn (*T*_1/2_ h)
50	>48	>48
10	>48	11.2 ± 1.4
1	29.7 ± 5	10.3 ± 1.1
0	30 ± 0.9	21.3 ± 1.9
[HSP10] (μM)	WT α‐syn 1% seed	A30P α‐syn 1% seed
50	>48	>48
10	21.3 ± 1.7	11.7 ± 0.8
1	8.7 ± 0.8	21.5 ± 1.6
0	9.3 ± 0.8	13.8 ± 0.4
0 (no seed)	23.6 ± 1.7	28.5 ± 3.5
Average no HSP10, no seed	26.8 ± 3.7	24.9 ± 4.6

**FIGURE 2:**
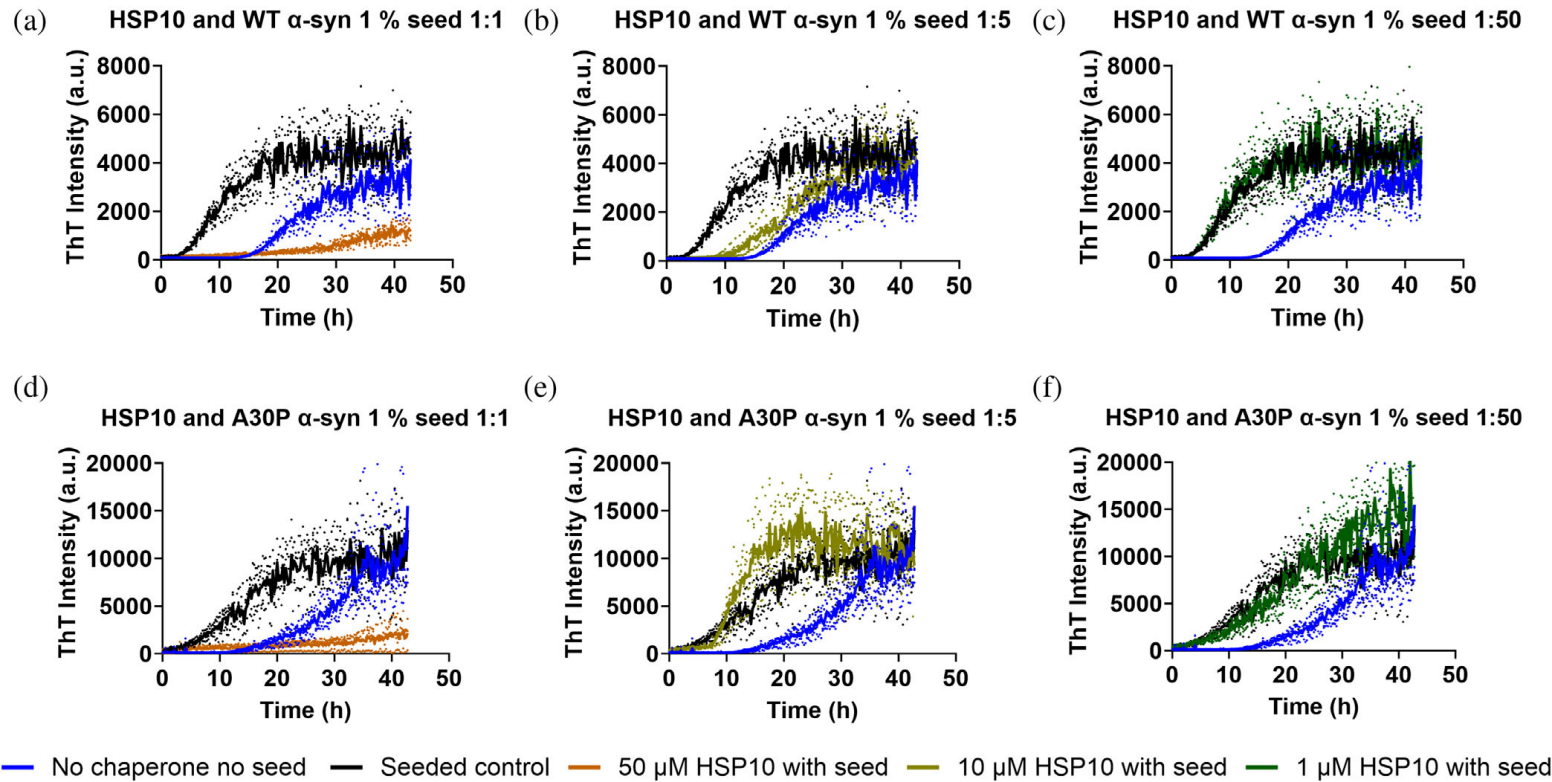
Fibril formation kinetics by ThT fluorescence during seeded conditions with different HSP10: α‐syn ratios. Herein, 50 μM WT α‐syn and A30P α‐syn were aggregated in the absence or presence of HSP10 at concentrations of: 50 μM (a) and (d), 10 μM (b), and (f) and 1 μM (c) and (f). Unseeded aggregation kinetics without chaperone are shown in the blue traces in every graph. Black traces show α‐syn seeded with 1% seed. The kinetics was conducted in a half area 96 well plate (corning 3881) at 37°C with shaking in between the measurement. All measurements were done in 4 replicates.

Under seeded conditions, that is, with added 1% preformed fibrils (PFFs), HSP10 again inhibited the fibril formation for WT α‐syn at the ratios of 1:1 (Figure 2a and Table 1) and to some extent at 1:5 (Figure 2b). The inhibition activity was abolished at a ratio of 1:50 (Figure 2c). When assaying seeded conditions for A30P α‐syn a complete inhibition of aggregation was again observed at the 1:1 ratio (Figure 2d), whereas tendencies of acceleration as evident from a steepened growth phase could be observed at the 1:5 ratio (Figure 2e and Table 1). This suggest that acceleration of fibril formation in this condition is both driven by the preformed seeds and the presence of the chaperone.

### 2.2 HSP10 binding of α‐synuclein fibrils

**FIGURE 3:**
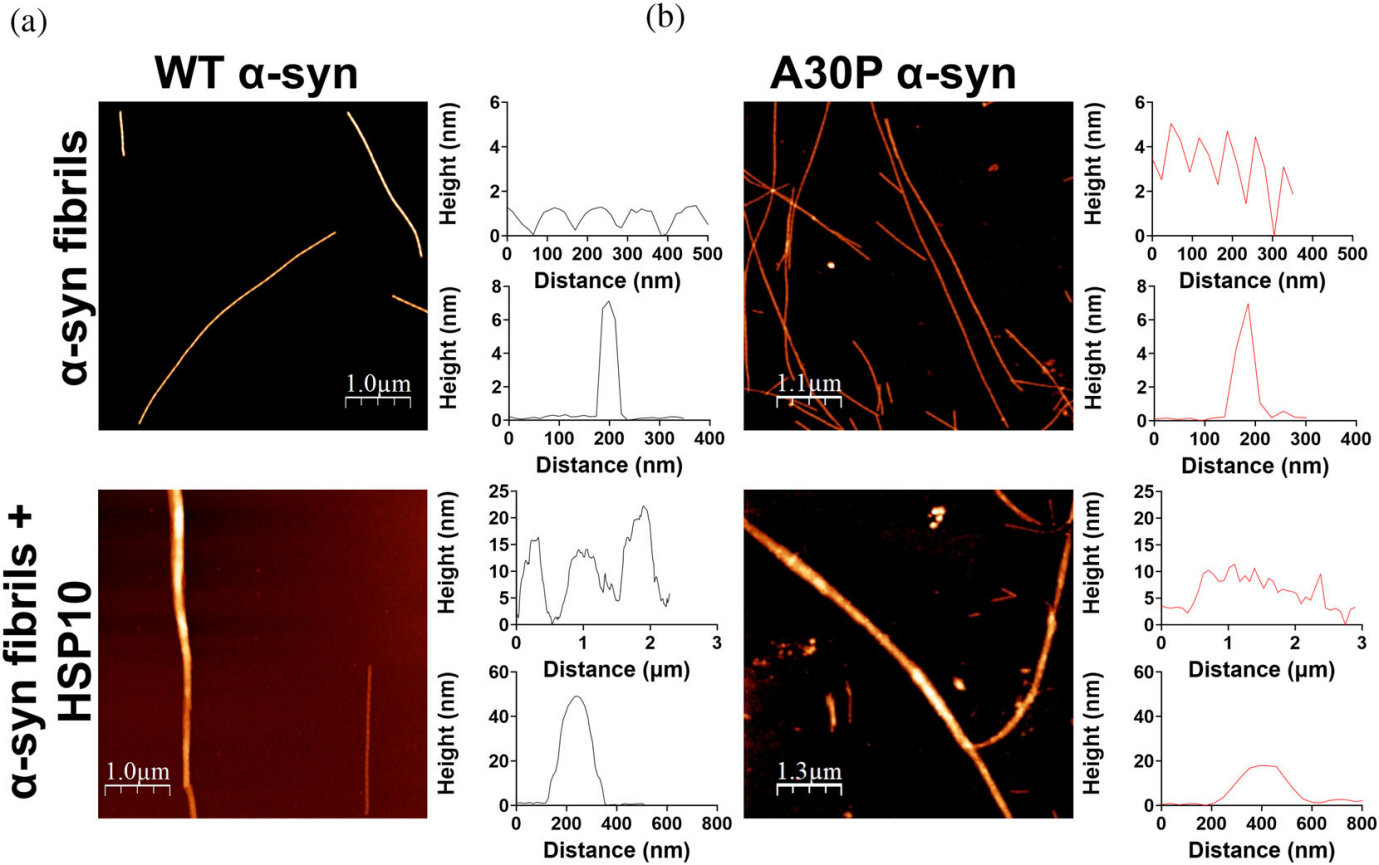
Characterization by AFM of HSP10 binding to WT and A30P α‐syn fibrils. (a) AFM images of WT α‐syn fibrils in absence (top), and presence of HSP10 (bottom). Periodicity and cross section (height) of amyloid fibrils. (b) Corresponding data for A30P α‐syn fibrils.

**FIGURE 4:**
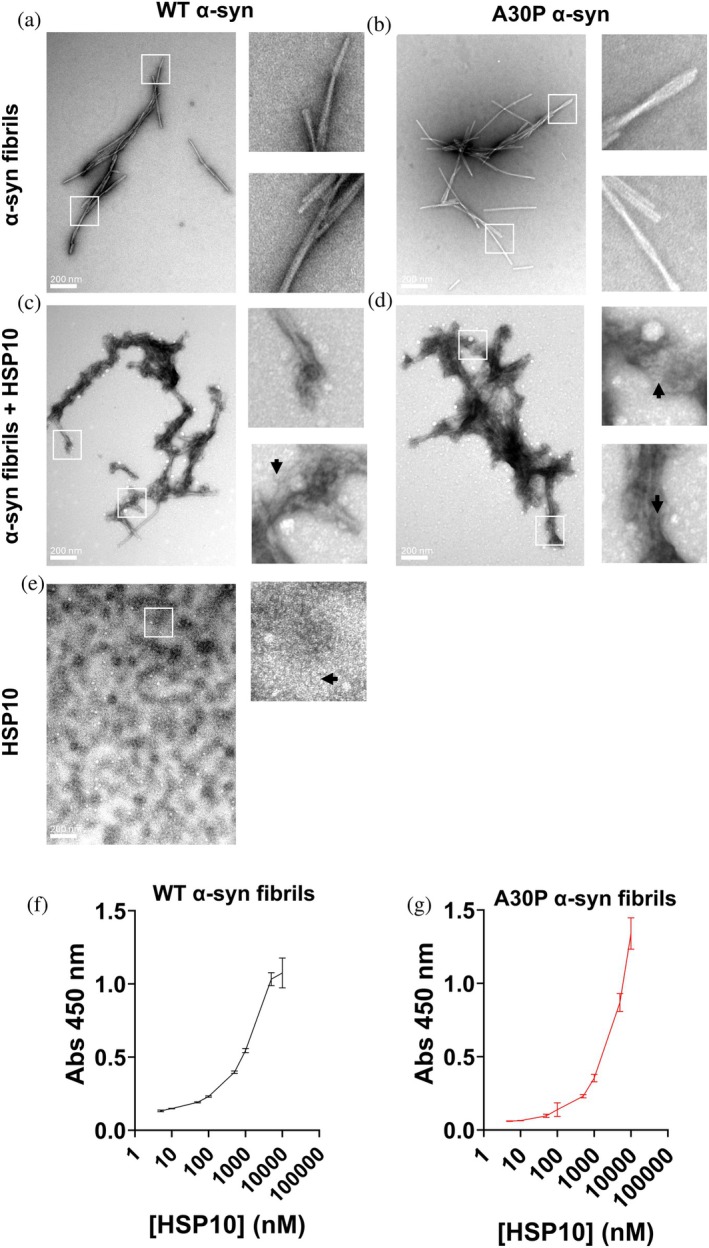
Characterization HSP10 binding to WT and A30P α‐syn fibrils. Negative stain TEM images of (a) WT α‐syn fibrils, (b) A30P α‐syn fibrils, (c) WT α‐syn fibrils with added HSP10 (1 μM), (d) A30P α‐syn fibrils with added HSP10 (1 μM), and (e) HSP10 chaperone alone (5 μM). Scale bars indicate 200 nm. The white frames show zoomed in regions depicted to the right of each image and the arrows indicate top views of HSP10 heptamers. (f) and (g) ELISA binding assay of HSP10 binding to plates coated with α‐syn fibrils, where anti‐6‐His antibody with affinity for the His‐tag on HSP10 was used for detection. The assay was run in triplicates and error bars represent the standard deviation (SD). Data depicted in black for WT and in red for A30P α‐syn fibrils, respectively.

The chaperone interaction with α‐syn was further investigated in order to identify if the inhibited and accelerated aggregation kinetics were dictated by interactions with monomeric α‐syn or fibrillar α‐syn. AFM was first utilized to investigate if the chaperone binds WT and A30P α‐syn preformed fibrils in a 1:1 ratio (10 μM α‐syn (on a monomer basis) + 10 μM HSP10) (Figure 3a,b). In the absence of chaperone WT α‐syn fibrils exhibited a periodicity of approximately 110 nm and a cross‐section of approximately 7 nm (Figure 3a). The A30P α‐syn fibrils showed a periodicity of approximately 70 nm and a cross‐section of approximately 7 nm (Figure 3b). When HSP10 was added to preformed WT or A30P α‐syn fibrils some sections showed a rather dramatic increased cross section to around 50 nm for WT α‐syn fibrils (Figure 3a) and 17 nm for A30P α‐syn fibrils (Figure 3b). Measurements along the axis revealed a 15 nm height fluctuation for WT fibrils and 5 nm for A30P fibrils. In addition, the periodicity showed substantially altered patterns with broadening to 700–800 nm for HSP10 bound WT α‐syn fibrils (Figure 3a) and a wide non‐periodic pattern for A30P α‐syn fibrils (Figure 3b). To induce such large geometric differences, layers of multiple HSP10 chaperones need to cover the fibrils. To further confirm HSP10 binding to α‐syn fibrils, we complemented the AFM analysis with negative stain transmission electron microscopy (TEM). Preformed fibrils were generated from 50 μM monomeric WT and A30P α‐syn that were incubated for 48 h during shaking in a plate reader as in Figure 1. In order to reduce the HSP10 coverage we added 1 μM of HSP10 chaperone and in the control sample only buffer vehicle (minor dilution with the same buffer). The final ratio was 40:1 (40 μM α‐syn (on a monomer basis) + 1 μM HSP10). These samples were incubated side by side for 24 h in room temperature before preparing TEM grids. Fibrils with buffer vehicle added displayed symmetric fibrous structures with a tendency for fibril‐fibril associations (Figure 4a,b). The overall morphology of fibrils from WT and A30P α‐syn were similar. However, when HSP10 was added, the fibrils showed dramatic morphological differences, displaying almost complete coverage of HSP10 chaperone molecules all over the fibril surfaces (Figure 4d,c). Individual HSP10 chaperone molecules were distinguishable but were difficult to identify (cf. Figure 4a,b inset with Figure 4c,d and with Figure 4e). Even at this low ratio of HSP10 it was apparent that HSP10 formed multilayered coverage over the fibrils verifying the results from the AFM analysis. The changed appearance of the fibrils was suggestive of fibril structural changes but the dense HSP10 coverage of the fibrils precluded evaluation of such potential fibril structural rearrangement. We can however clearly state that HSP10 was effectively bound to preformed fibrils of both WT and A30P α‐syn.

To further compare the affinity of HSP10 to α‐syn fibrils, we used an ELISA binding assay with HSP10 as primary binder towards sonicated immobilized α‐syn fibrils (Larsson et al., 2022). HSP10 was seen to clearly bind to the fibrils with an apparent dissociation constant (K_d_) of 1.45 μM towards WT α‐syn (Figure 4f). The binding of HSP10 towards A30P α‐syn did not yield saturation under the investigative condition; however, assuming a plateau was reached at the endpoint, the K_d_ would correspond to 2.9 μM (Figure 4g). Our obtained affinities were in accordance with previous reports of HSP10 affinity towards WT α‐syn fibrils using SPR (K_d_ = 5.27 μM; Szegő et al., 2019). The affinity measurements likely reflect an average K_d_ of the multiple layers of HSP10s bound to α‐syn fibrils. HSP10 showed a higher affinity towards the WT α‐syn fibrils compared to A30P fibrils, and WT fibrils were more densely covered by HSP10 as shown by AFM.

### 2.3 HSP10 binding of α‐synuclein monomers

**FIGURE 5:**
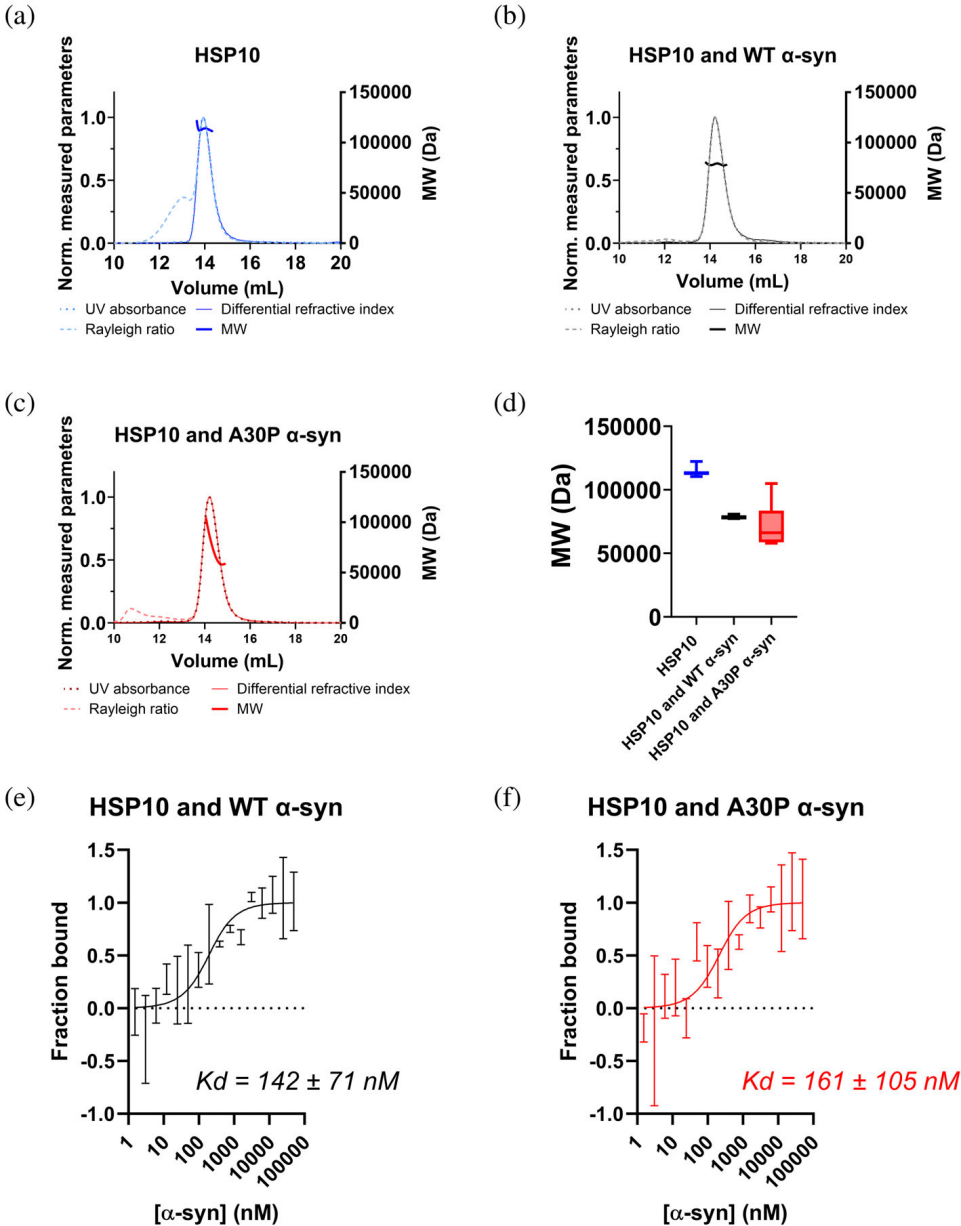
SEC‐MALS chromatograms with Differential refractive index, Rayleigh ratio, UV absorbance and MW (a) SEC‐MALS chromatogram of 80 μM HSP10. (b) HSP10 with WT α‐syn monomer at a 1:1 ratio (c) HSP10 with A30P α‐syn monomer at a 1:1 ratio. The molecular weight (MW) over the peak is shown as the thicker line from the corresponding color. (d) The distribution of MW for the HSP10 with and without monomeric WT α‐syn and A30P α‐syn. (e) MST binding curve of ^Cy5^HSP10 with WT α‐syn monomer and (f) MST binding curve of ^Cy5^HSP10 with A30P α‐syn monomer, with calculated apparent dissociation constants (K_d_).

To assess if and how HSP10 interacted with monomeric α‐syn we employed size exclusion chromatography small angle X‐ray scattering with multiangle light scattering (SEC‐SAXS/MALS), and batch SAXS measurements. Firstly, before adding α‐syn, we observed that pure HSP10 chaperone displayed an apparent molecular weight of 113.5 ± 2.1 kDa with SEC‐MALS at the main peak of the chromatogram (Figure 5a), and 113.7 kDa (106.9–127.5 kDa) with SAXS (Table 2). Given the sequence (HSP10 with a his‐tag on each subunit) we expected a molecular weight of 88.2 kDa for heptameric HSP10. This suggested that HSP10 heptamers showed tendencies for self‐association. Self‐association of HSP10 has previously been documented, both in the context of heptameric ring stacking (Luke et al., 2005) as well as a face‐to‐face tetradecameric structure formation (Roberts et al., 2003). It was also hypothesized that tetradecameric HSP10 could enclose misfolded HSP10 monomers (Roberts et al., 2003). It is also possible that HSP10 is bound to another protein; however SDS‐PAGE and sensitive staining of the gel showed pure HSP10 (Figure S1a). Our preparations of HSP10 revealed highly cooperative unfolding and refolding transitions (Figure S1b) showing that the chaperone can spontaneously and fully refold after temperature‐dependent denaturation used in our purification of the protein (Materials and methods). We therefore considered the HSP10 preparation to be pure. As the concentration of HSP10 was increased in SAXS measurements, *D*_max_ increased (Figure S2c) consistent with partial self‐interaction, and the flexibility appeared to increase as can be observed by the Kratky plot (Figure S2d). The scattering intensity curve of HSP10 alone revealed characteristic features for disc‐like proteins with a dip in the curve in between an angle of 1 and 2 nm^−1^ (Figure S2a). The curve looked similar to previously SAXS measurements conducted on the bacterial homologue GroES (Higurashi et al., 2003; Stegmann et al., 1998). Interestingly, as we added 1:1 α‐syn (WT or A30P) to HSP10, the apparent molecular weight decreased from 113.5 ± 2.1 kDa to 78.4 ± 0.8 kDa and 72.2 ± 15.1 kDa with SEC‐MALS (Figure 5a–d). The decreased apparent molecular weight of HSP10 compared to when alone indicated that a complex formed between HSP10 and monomeric α‐syn diverting HSP10 towards complex formation with α‐syn rather than partially self‐associated HSP10 chaperones. To estimate the affinity between HSP10 to monomeric WT and A30P α‐syn, we used Cy5‐labeled HSP10 and unlabelled α‐syn monomers for measurements with microscale thermophoresis (MST). The data showed very high HSP10 affinity for α‐syn with K_d_ = 140 ± 71 nM and 160 ± 105 nM for WT and A30P, respectively (Figure 5e,f).

**TABLE 2:** SAXS molecular weight and radius of gyration (*R*_*g*_) data.

Sample	[α‐syn] μM				
	0	10	21	41	83
WT α‐syn + HSP10 (kDa)	113.7	94.2	113.7	85.7	74.3
WT α‐syn + HSP10 Credibility Interval (kDa)	106.9–127.5	89.7–99.2	106.9–121.5	77.4–89.7	69.7–81.9
WT α‐syn + HSP10 *R*_*g*_ (nm)	3.969	3.837	3.802	3.792	3.796
A30P α‐syn + HSP10 (kDa)	113.7	101.0	94.2	109.1	94.2
A30P α‐syn + HSP10 Credibility Interval (kDa)	106.9–127.5	92.7–111.0	89.7–95.8	89.7–121.5	87.0–107.0
A30P α‐syn + HSP10 *R*_*g*_ (nm)	3.969	4.120	4.380	4.426	5.714

**FIGURE 6:**
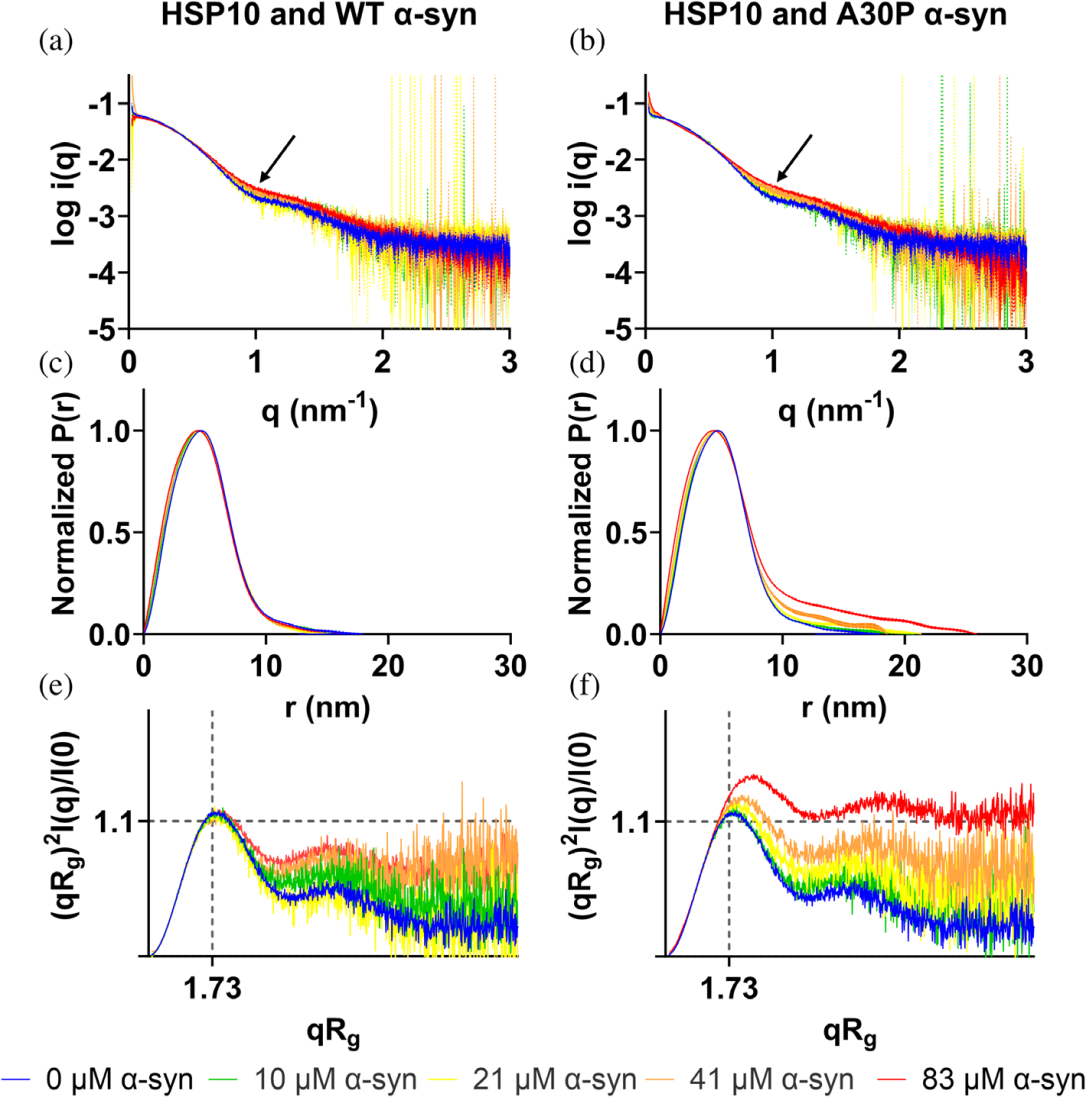
SAXS data with 11 μM HSP10 and α‐syn monomers in different concentrations. WT α‐syn (a), (c), and (e), A30P α‐syn in (b), (d) and (f). (a) and (b) show the logarithmic scattering plots. Arrows indicated the change in mid q region. (c) and (d) the distance distribution with real space *R*_*g*_ over concentration of α‐syn. (e) and (f) show the corresponding Kratky plots for HSP10 with either increasing concentration of WT α‐syn or A30P α‐syn. Specific details of the SAXS experiments are found in Tables S1–S6 (Trewhella, et al. 2023) and the Guinier plots in Figure S3.

We then performed a titration of different α‐syn monomer concentrations to a constant concentration of 11 μM HSP10 and monitored the SAXS profiles (Figure 6). As a first observation the molecular weight of HSP10 decreased at equimolar concentrations of α‐syn (Table 2) in correlation with the SEC‐MALS data (Figure 5). Notably at 1:1 ratio of HSP10 (11 μM) and WT and A30P α‐syn (10 μM) the molecular weight correlated better than for SEC‐MALS with a theoretical molecular weight of a 1:1 complex (102.7 kDa) (Table 2). As the monomer concentration of α‐syn increased the scattering curve changed its scattering profile in the mid‐q range (~1 nm^−1^) indicating that the shape of the chaperone changed as the concentration of α‐syn increased (marked with arrow in Figure 6a,b). No change was seen in P(r) when WT α‐syn was titrated to 11 μM HSP10 (Figure 6c). In contrast to this result, the maximum distance increased for HSP10 with increasing concentrations of A30P α‐syn (Figure 6d and Table 2). At 83 μM A30P α‐syn the *P*(*r*) showed a *D*_max_ up 25 nm suggesting induction of very large complex formation likely reflecting initiation of aggregation, that is, oligomerization of A30P α‐syn, consistent with accelerated fibril formation by the ThT assay at a similar HSP10:α‐syn ratio (here being 1:7.5). Another large difference between HSP10 with WT α‐syn and A30P was seen in the unfoldedness/flexibility of the system in the Kratky plot with a plateau at higher q values (Figure 6e,f). The presence of A30P α‐syn added flexibility to the system. This was to some extent observed for WT α‐syn (Figure 6e), but was more pronounced for A30P α‐syn, especially at the highest concentration (Figure 6f). This effect was also influenced by α‐syn monomers being observed at higher α‐syn concentrations, where A30P α‐syn also in the absence of HSP10 started to aggregate (Figure S4).

## 3 DISCUSSION

The chaperonins HSP60/HSP10 are highly expressed in the central nervous system and are essential for healthy proteostasis and likely plays a pivotal role in neurodegenerative disease. Interestingly HSP10 is even more abundant than HSP60 in the brain which encouraged us to study its independent function as a chaperone for amyloid fibril formation (Larsson et al., 2022). HSP10 was previously shown to both have an inhibitory effect and aggregation acceleration effect when present during fibril formation of Aβ1‐42 (Larsson et al., 2022). The ability of HSP10 to interact with different client proteins would be important for HSP10 to act as a universal chaperone to uphold proteostasis with and independently of HSP60. α‐syn is one of the most abundant proteins of the brain, particularly in neurons (Moors et al., 2022; Sulzer & Edwards, 2019). The protein is predominantly located in the cytoplasm and at synapses, likely in secretory vesicles (Sulzer & Edwards, 2019). HSP10 is the most abundant protein of mitochondria (Morgenstern et al., 2021). α‐syn fibril induced neuronal mitochondrial dysfunction in vivo in mice has recently been demonstrated (Geibl et al., 2024). Our in vitro biophysical studies in this report were hence motivated by HSP10 involvement in mitochondrial proteostatic response to α‐syn fibril infiltration in synucleinopathy. The HSP10 interaction with α‐syn, has previously been investigated showing that α‐syn aggregate entrapment of HSP10 lead to cytotoxicity and that overexpression of HSP10 mitigated α‐syn pathology (Szegő et al., 2019). That study reported HSP10 interference with WT α‐syn fibril formation and found less evidence for affecting fibril formation of A30P α‐syn. However, the in vitro experiments reported by Szegő and coworkers (Szegő et al., 2019) were performed with surprisingly low α‐syn concentrations. The critical concentration for unseeded WT α‐syn fibril formation is approximately 10 μM (Afitska et al., 2019), and we therefore worked in and above that concentration range. The findings from our study clearly showed that HSP10 can inhibit fibril formation of both WT and A30P α‐syn at sufficient HSP10 concentrations. Interestingly, however, it was very clear that specific differences of the α‐syn client protein can dictate the threshold between inhibition and acceleration of fibril formation. WT α‐syn aggregation was efficiently inhibited by the presence of HSP10. At a ratio of 1:5 (HSP10:α‐syn) the aggregation inhibition was still complete, with no amyloid formation detected in the ThT assay. This was not the case for A30P α‐syn. Here, HSP10 showed the dual effect of inhibition at equimolar conditions while acceleration of amyloid formation occurred at the ratio of 1:5. Peculiarly, this is a very high HSP10:client ratio. Our previous studies with Aβ1‐42 found acceleration of fibril formation at a 100‐fold lower ratio of chaperone versus client protein (Larsson et al., 2022). Hence this effect should not be related to spontaneous structural changes of HSP10 but appeared to be a client specific effect.

Notably, under pre‐seeded conditions, HSP10 could still prevent amyloid formation of WT α‐syn as well as with the A30P mutant at a 1:1 ratio of HSP10 and α‐syn. However, at the ratio of 1:5 for the A30P mutant, the inhibitory effect of HSP10 was completely lost. Not only that, but a slight acceleration of fibril growth was observed when compared to the seeded control, suggesting a dual acceleration of α‐syn for seeds and the chaperone.

**FIGURE 7:**
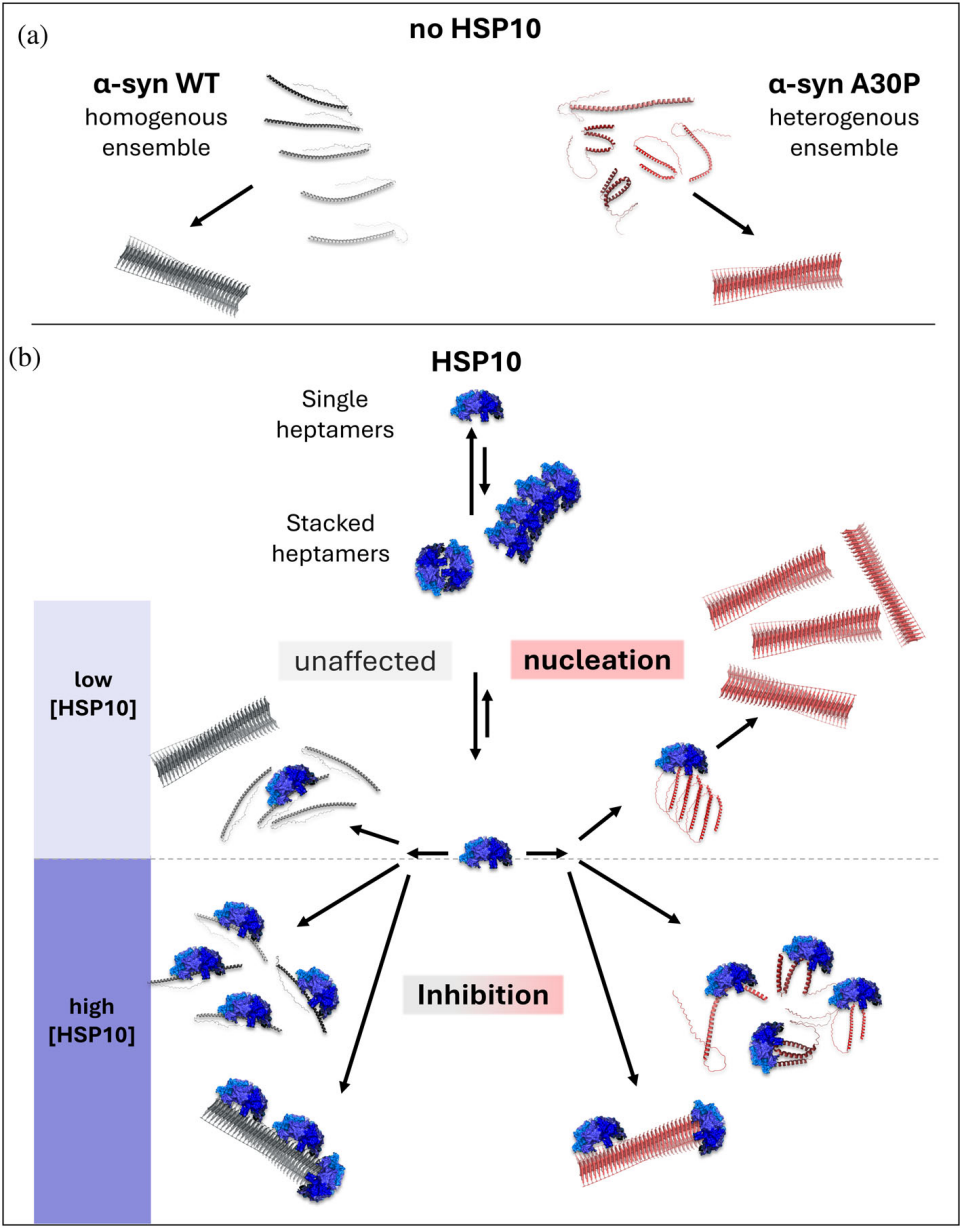
Illustration of the mechanisms of HSP10 mediated inhibition of fibril formation of WT and A30P α‐syn and acceleration of A30P α‐syn under different concentrations of HSP10. (a) Structure models of WT and A30P α‐syn from Alphafold3 (AP3) (https://alphafoldserver.com/) displaying a more homogenous conformational ensemble for α‐syn monomers of WT than for A30P due to the point mutation. Note that α‐syn without lipids is an intrinsically disordered protein so AP3 overestimates the amount of α‐helical structure in the N‐terminal domain which may be deceiving. (b) Fibril formation of α‐syn in the presence of various concentrations/stoichiometry of HSP10. At low concentrations (top) fibril formation of WT appears unaffected by HSP10 while multiple A30P monomers bind HSP10 and form a chaperone mediated oligomeric nucleus accelerating fibril formation. At higher HSP concentrations, HSP10 inhibits α‐syn fibril formation due to binding and sequestering monomers and fibrils with the higher efficiency towards WT α‐syn.

We have previously shown that HSP10 bound with high affinity to preformed fibrils of Aβ1‐42 (Larsson et al., 2022). Here we showed that the promiscuous nature of HSP10 enabled the chaperone to also bind α‐syn preformed fibrils, showing a dense and multilayered coverage of chaperones over the fibril surface. This is consistent with inhibition of seeded fibril formation by blocking secondary nucleation preventing surface mediated oligomerization. The binding affinity to fibrils was similar between WT and the A30P α‐syn, with a tendency for better affinity towards WT α‐syn fibrils. WT α‐syn fibrils were also more heavily covered by HSP10 than A30P α‐syn fibrils. The stronger fibril binding may explain the better inhibitory activity of HSP10 for WT compared to A30P α‐syn. However, this suggests that the affinity for the fibrillar material does not dictate the acceleration of fibril formation that was observed for the A30P mutant. Neither does the HSP10 affinity for α‐syn monomers differ between WT and A30P. Therefore, where that interaction of HSP10 differed between the WT and the A30P mutant is in the dynamic interaction with monomers as shown by SAXS. The presence of monomeric A30P appeared to impact both the MW of the chaperone complex as well as the radius of gyration which at higher concentrations of A30P reflected an initiation of aggregation, that is, oligomerization of A30P in accordance with the observed acceleration of fibril formation kinetics. Aggregation of α‐syn is catalyzed by the non‐amyloid beta component (NAC) sequence. This region contains similar segments that we have previously hypothesized to be important for HSP10 association. More specifically the region 67–71 GGAVV found in NAC is similar to our hypothetical region of interest when HSP10 interacted with Aβ and PrP (Larsson et al., 2022). Curiously, familial synucleinopathies such as familial Parkinson's disease (fPD) almost exclusively stem from mutations in the earlier N‐terminal region not part of the NAC (Meade et al., 2019). This region shows high homology with the chaperone 14‐3‐3 (Ostrerova et al., 1999), and appears to be important for the intrinsic chaperone activity of α‐syn and its involvement in stabilizing SNARE complex formation (Burré et al., 2010). Our data suggest that the A30P mutation increases the binding efficacy of multiple monomers towards the HSP10 chaperone which leads to accelerated aggregation under sub stoichiometric ratios of HSP10 compared to α‐syn. The A30P mutation likely induces a kink in the helical folding propensity of α‐syn, as predicted by alpha‐fold modeling (Figure 7a), rendering a heterogenous conformational ensemble of bent or curved monomers which have a higher propensity towards HSP10 mediated oligomerization than WT α‐syn. We found that the rates of spontaneous fibril formation of A30P and WT α‐syn varied quite substantially (mostly for A30P) and hence the average *T*_1/2_ was overall similar between the two α‐syn variants (Table 1). There have been diverging reports on the effect of the A30P α‐syn mutation in comparison with WT α‐syn regarding aggregation and fibril formation rates. A30P has been reported to slow down fibril formation (Lemkau et al., 2012) or not affect the aggregation rates (Conway et al., 2000). The majority of studies argue for acceleration of aggregation rates (Lashuel et al., 2002; Li et al., 2001, 2002), while fibril formation (ThT positive) appears slower (Lemkau et al., 2012; Li et al., 2001) likely because A30P promoted protofibril formation (Lashuel et al., 2002). A recent report by Ohgita (Ohgita et al., 2022) reported a thorough kinetic analysis based on ThT fluorescence and concluded that the A30P mutation increased the aggregation rate and to some extent the fibril formation rate of α‐syn by decreasing the enthalpic barrier for nucleation. A decreased enthalpic barrier for nucleation of the mutant is consistent with our data of specific nucleation enhancement for A30P α‐syn monomers by binding to HSP10. Our data suggest that HSP10 directs conversion of A30P α‐syn into fibrils, rather than being trapped into off‐pathway protofibrils/oligomers, akin to fibril nucleation by a preformed fibril seed. Such a mechanism would be compatible with previous reports showing that the A30P mutation rendered α‐syn prone to entrapment in oligomers, off‐pathway towards fibrils (Tosatto et al. 2015). HSP10 binding hence steered the path of A30P towards fibril converting oligomers.

Self‐association of HSP10 heptamers is a previously documented process; this has both been seen as stacking of heptameric HSP10 and formation of tetradecameric balls of the chaperone (Luke et al., 2005; Roberts et al., 2003). Depending on the context, chaperone self‐association can be an intended mechanism to increase the soluble concentration of the chaperone. Small heat shock proteins have been shown to self‐associate and form oligomers that are essential for the chaperone function (Haslbeck & Vierling, 2015; Lelj‐Garolla & Mauk, 2006). Self‐association can also be an early initiation of aggregation leading to loss‐of‐function of the chaperone. The decrease in MW of HSP10 when monomeric α‐syn is introduced would suggest that the self‐association of HSP10 is a temporary state that is interrupted when the demand on heptameric chaperones increases. In this context, we find it likely that the macromolecular self‐assembly state is a temporary state, similar to how small heat shock proteins oligomerize.

In summary, HSP10 shifts from self‐interactions towards heptameric active chaperone in the presence of client protein (α‐syn). At a sufficient stoichiometric ratio, HSP10 interacts with α‐syn fibrils and monomers and thereby efficiently inhibits fibril formation (Figure 7b, bottom). The A30P mutation specifically increases the propensity for α‐syn monomer/oligomer recognition of the chaperone. Hereby, multiple A30P α‐syn monomers can bind to HSP10 at sub‐stoichiometric chaperone concentrations, shifting the inhibitory activity towards an acceleration of fibril conversion by chaperone‐mediated oligomerization and fibril nucleation (Figure 7b, top).

## 4 CONCLUSION

The chaperone activity of HSP10, independent of HSP60, is a novel finding that complements our understanding of proteostasis. The switch between inhibition and acceleration of amyloid fibril formation by this chaperone is a paradoxical mechanism but it appears to be a general function as we have observed this switch for Aβ1‐42 and PrP90‐231 (Larsson et al., 2022) and here for A30P α‐syn. The function appears to be a consequence of HSP10:client stoichiometry, HSP10 amyloid fibril and monomer interaction, and is context dependent. It is clear that the acceleratory window at low stoichiometric ratios of HSP10 to client protein is highly dependent on the client protein conformational properties, since we observed fibril formation acceleration of A30P α‐syn, but not for WT α‐syn. Our findings are well in agreement with the pathological properties of A30P α‐syn and may have implications for the onset of A30P α‐syn fibril formation under chaperone exhausted conditions during proteostatic stress.

## 5 MATERIALS AND METHODS

### 5.1 HSP10 purification

Recombinant human HSP10 purification followed the previously described method found in JNK, Larsson et al. (2022). In short, HSP10 was produced in *Escherichia coli* BL21, transformed with a plasmid expressing human HSP10 with an N‐terminal hexa‐His tag. To achieve a pure yield the protein lysate was heated to 85°C to remove most impurities followed by nickel affinity chromatography (NTA‐agarose) and size exclusion chromatography using a Superdex 75 gel filtration column in PBS pH 7.4 buffer. The pure protein was dialysed in TBS buffer pH 7.6 and the concentration was measured using in a spectrophotometer with the extinction coefficient of 5960 M^−1^ cm^−1^ at 275 nm. Purity was confirmed using SDS‐page and staining with colloidal Coomassie G‐250 which has comparable sensitivity to silver staining (Kang et al., 2002).

### 5.2 α‐Synuclein purification

Human WT and A30P proteins were expressed in Escherichia coli. In short, plasmids for WT and A30P variants were transformed into BL21 (DE3) (Novagen) cells. The bacteria were first grown to an OD_600_ of 0.8 in Luria broth (LB) containing 100 μg/mL carbenicillin at 37°C and then induced with 1 mM IPTG (isopropyl β‐d‐1‐thiogalactopyranoside) and grown overnight at 25°C post induction. The cells were harvested and lysed by sonication on an ice bath in 20 mM Tris–HCl buffer pH 8.0 in the presence of protease inhibitor cocktail (Roche). The lysate was then heat treated at 90°C in a water bath for 10 min followed by centrifugation at 15000×*g* for 30 min. The supernatant was filtered through a 0.2 μm filter and loaded onto a pre‐equilibrated 5 mL HiTrap Q FF anion exchange column (GE Healthcare). The α‐syn proteins were eluted by a linear gradient with 1 M NaCl in 20 mM Tris–HCl buffer pH 8.0. The eluted protein was run on a 4–12% SDS‐PAGE and fractions containing the protein of interest were pooled and concentrated with Amicon Ultra‐15 10 K centrifugal filter units (Millipore). The concentrated protein was loaded and retrieved from a pre‐equilibrated Hiload 16/600 Superdex 75 pg. column (GE Healthcare) with 20 mM Tris‐sulfate buffer pH 7.4. For all purified α‐syn variants, the sample purity was confirmed by a single band on SDS‐PAGE gel, a single elution peak in size exclusion chromatography, and by mass spectrometry. Fractions containing pure protein were pooled and snap frozen in liquid nitrogen and stored at −80°C. The concentration of WT and A30P was determined using extinction coefficient 5960 M^−1^ cm^−1^ at 280 nm.

Frozen fractions were thawed and sterile filtered using a 0.22 μm PVD filter. The protein was then loaded on a pre‐equilibrated 10/300 Superdex 75 increase column (Cytiva) with TBS buffer pH 7.6. The fractions containing the monomeric protein were collected and concentrated using an Amicon Ultra Centrifugal Filter, 10 kDa MWCO (Millipore). The concentration was determined using the extinction coefficient 5960 M^−1^ cm^−1^ at 280 nm for both WT and A30P α‐syn. This protein was used for all the assays.

### 5.3 Fibril formation kinetic ThT‐assay

ThT‐assays were conducted in TBS 0.02% sodium azide pH 7.4. α‐syn (50 μM) was mixed with HSP10 and the different conditions were then loaded in a half area 96 well plate (corning 3881) with 10 μM ThT and a 3 mm glass bead. α‐syn seeds were prepared by sonicating fibrils using a Branson sonifier with a micro‐tip with the settings 1 min on 1 min off 30% amplitude for a total of 6 min on. The plate was incubated in a CLARIOstar plate reader at 37°C. The ThT intensity was measured every 15 min (excitation 440 emission 480) and was used within each cycle where 300 s of orbital shaking was conducted at 600 rpm. The data was plotted using GraphPad prism 10. Half‐time of fibril conversion (*T*_1/2_) was extracted by fitting the individual kinetic traces to a transformed Boltzmann function in Origin 2024.

### 5.4 Atomic force microscopy (AFM)

Wild type or mutant A30P α‐syn amyloid fibrils were incubated overnight with HSP10, at room temperature, equimolar (10 μM α‐syn fibrils + 10 μM HSP10). Samples were deposited onto freshly cleaved mica. After 30 min, the mica samples were rinsed 3–4 times with MilliQ water then dried completely under a gentle stream of nitrogen gas. The images were captured using an NTEGRA Prima setup at a resonance frequency around 180 kHz, using a gold‐coated single crystal silicon. Images of 256 pixels were captured. Images were analyzed using WSxM 5.0 software.

### 5.5 Transmission electron microscopy (TEM)

Negative stain (TEM) was performed as follows: Preformed fibrils were generated from 50 μM monomeric WT and A30P α‐syn that were incubated for 48 h during shaking in a plate reader. 1 μM of HSP10 chaperone, and in the control sample only buffer vehicle (minor dilution with the sample buffer) was added to the fibrils resulting in a final α‐syn:HSP10 ratio of 40:1 (40 μM α‐syn (on a monomer basis) + 1 μM HSP10). Samples of 5 μM HSP10 chaperone alone were made as reference. The samples were incubated side by side for 24 h in room temperature before preparing TEM grids. Five microliters of sample was applied to carbon/formvar coated 400 mesh copper grids (Ted Pella Inc.) and was absorbed for 2 min. Excess sample was removed by a filter paper. Grids were washed with 5 μL milliQ water and were stained for 30 s with 2% uranyl acetate. Grids were blotted dry and were dried in room temperature. TEM imaging was performed using a Jeol JEM1400 Flash TEM microscope operated with an acceleration voltage of 80 kV.

### 5.6 Chaperone binding assay

Chaperone binding assay was conducted with α‐syn fibrils α‐syn. Preformed fibrils were sonicated using a Branson sonifier with a micro‐tip (1 min on, 1 min off, Amp 30% for 6 min on). The fibrils were then diluted in a 15 mM Na_2_CO_3_, 35 mM NaHCO_3_ buffer (pH 9.6) to a final concentration of 2.5 μg/mL. 50 μL was loaded on a 96 well high binding plate (Corning 9018) and incubated overnight. The liquid in the wells was removed and the plate was washed four times with 100 μL PBST 0.05% tween. This washing was repeated in between every step of the binding assay. 50 μL of 2% BSA in PBS was added to each well as a blocking agent and the plate was incubated at room temperature for 1 h. HSP10 chaperone (with a 6×His‐tag) placed in blocking solution at different concentrations was added to the wells, and the plate was incubated for 2 h at room temperature. 50 μL rabbit polyclonal anti 6×His (Ab1187) antibody in blocking solution was added to each well, and the plate was incubated at room temperature for 1 h. 50 μL TMB solution was added to develop the plate, and the reaction was stopped with 5 μL 0.18 M H_2_SO_4_. Absorbance at 450 nm was measured in a Tecan Safire 2.

### 5.7 Microscale thermophoresis (MST)

1 mL of HSP10 (1 mg/mL) dissolved in PBS buffer (pH 7.4) was labeled by addition of 10 μL of a 15 mg/mL Cy5 NHS‐ester (Lumiprobe) dissolved in DMSO, to a final concentration of 225 μM Cy5 NHS‐ester. The labeling reaction was incubated for 60 min at room temperature whereafter the reagent was quenched by addition of 100 mM Tris–HCl pH 7.5. The labeled protein was purified by a desalting G25‐gelfiltration PD10 column (GE Healthcare) using PBS as running buffer separating ^Cy5^HSP10 from free Cy5 dye. The degree of labeling of ^Cy5^HSP10 was determined by absorbance for Cy5 and HSP10 (250,000 M^−1^ cm^−1^ at 649 nm and HSP10 5960 M^−1^ cm^−1^ at 275 nm) and was approximately 1.2 Cy5‐dyes per HSP10 heptamer. Samples for MST were prepared with 100 nM of ^Cy5^HSP10 and either WT α‐syn or A30P. The concentration of α‐syn was between 50,000 nM and 1.5 nM. The samples were incubated at 37°C for 1 h prior to measurement. MST measurements were conducted in a Monolith NT.115 (Nanotemper) instrument at 37°C with the excitation power at 30% and the MST power at 40%. Two independent experiments were performed for each α‐syn variant. The binding affinity of HSP10 to α‐syn was estimated and evaluated using the MO. Affinity Analysis v2.3 software. A standard fitting model derived from the law of mass action was used to determine the K_d_ for both α‐syn variants (Jerabek‐Willemsen et al., 2011).

### 5.8 Small angle X‐ray scattering (SAXS) in batch

Samples were dialysed into TBS pH 7.6 buffer and mixed in the ratios described above. Batch runs were conducted at EMBL‐P12‐bioSAXS beamline (PETRAIII, Desy, Hamburg). Batch data were analyzed using PRIMUS ATSAS 4.1.0 software (Manalastas‐Cantos et al., 2021). Guinier analysis and inverse Fourier transformation were used to determine the structural parameters found in Tables 2, S1 and S2.

### 5.9 Size exclusion chromatography combined with multi‐angle light scattering and SAXS (SEC‐MALS/SAXS)

SEC‐MALS/SAXS was performed at EMBL‐P12‐bioSAXS beamline (PETRAIII, Desy, Hamburg). HSP10 was incubated with α‐syn in a 1:1 ration (80 μM HSP10 and 80 μM α‐syn) in 37°C for 1.5 h prior to injection. 90 μL protein was injected on a pre‐equilibrated 10/300 Superdex 200 increase column (GE Healthcare) with TBS pH 7.6. Flow rate was set to 0.8 mL/min and experiments were carried out at 20°C. MALS data was measured on a Mini‐Dawn TREOS detector and an optiLab T‐rex refractometer. The concentration was determined using Ri with the dn/dc value of 0.1850 mL/g. The data was analyzed in ASTRA6 from Wyatt technology.

### 5.10 Thermal unfolding and folding by differential scanning fluorescence (Nano‐DSF)

Thermal stability and folding reversibility of HSP10 was assessed by nano‐DSF using a Prometheus NT‐48 instrument (Nanotemper). 10 μM HSP10 in PBS pH 7.4 buffer was loaded into capillaries, the thermal scan was performed from 20 to 85°C and cooling within the reverse range using a ramp rate of 0.5°C/min and recording the 330 and 350 nm intrinsic fluorescence signal. Samples were assayed in triplicate. The inflection points of the first derivative of the fluorescence change monitored at 350/330 nm were denoted as the unfolding temperature and refolding temperature, respectively.

## AUTHOR CONTRIBUTIONS

**Johan N. K. Larsson:** Conceptualization; investigation; writing – original draft; formal analysis; visualization; methodology; data curation; writing – review and editing. **Ranjeet Kumar:** Methodology; resources. **Fiamma Ayelen Buratti:** Investigation. **Sofie Nyström:** Supervision; funding acquisition; visualization; methodology; writing – review and editing. **Pernilla Wittung‐Stafshede:** Funding acquisition; writing – review and editing; supervision; conceptualization. **Per Hammarström:** Conceptualization; investigation; writing – review and editing; supervision; methodology; project administration; funding acquisition; formal analysis.

## FUNDING INFORMATION

The study was funded by grants from the Swedish Brain Foundation (ALZ2019‐0004 and ALZ2022‐0004 Per Hammarström, Sofie Nyström), Swedish Research Council (VR 2023‐03427 PWS; 2023‐03931 Per Hammarström), KAW Scholar (PWS), Konung Gustav V and Drottning Victorias Foundation (Per Hammarström), and Stiftelsen för Parkinsonforskning (Per Hammarström).

## Supporting information


**DATA S1.** Supporting Information.

## Data Availability

The data that support the findings of this study are available from the corresponding author upon reasonable request.
