# Supplementary material for: Heat shock protein 10 as a chaperone modulating α‐synuclein amyloid fibril formation
Source: Protein Sci. 2026 Jan 20;35(2):e70452. doi: 10.1002/pro.70452 (PMC12817295; doi:10.1002/pro.70452)
Supplement: Supplementary file 1 — DATA S1. Supporting Information. [file PRO-35-e70452-s001.docx]

**Supplementary Figures**


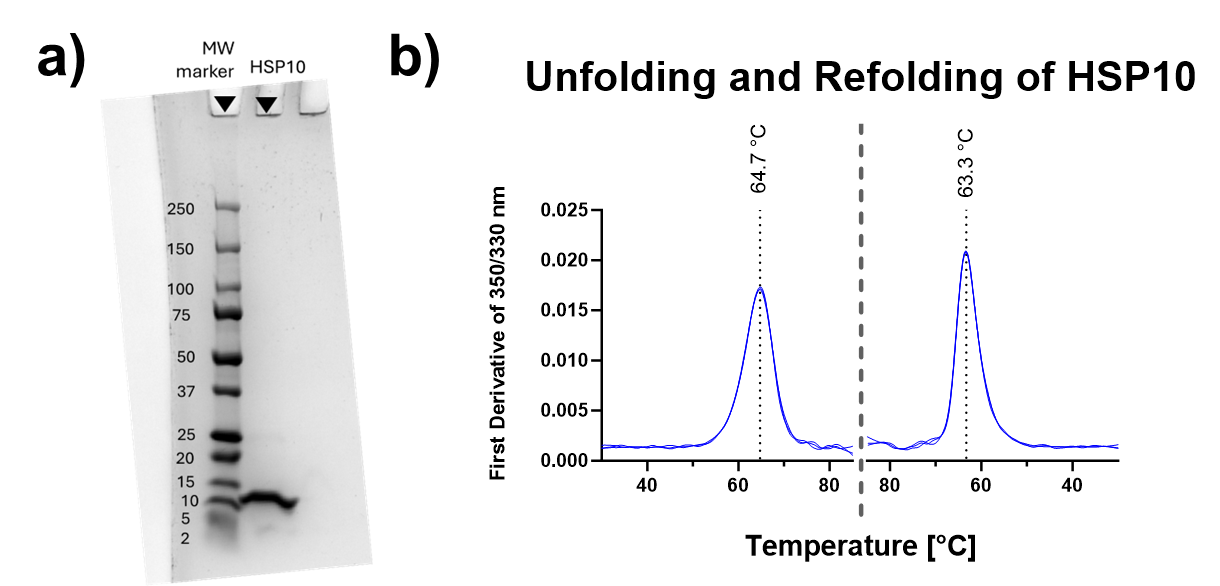


**Figure S1. a)** SDS-PAGE gel of HSP10 stained with sensitive colloidal Coomassie G-250 showing purity and expected molecular weight of 12.6 kD of the denatured HSP10 monomer. **b)** Thermal unfolding and refolding of 10 µM HSP10 in PBS buffer. The assay was conducted in the Nanotemper nanoDSF Prometheus NT-48, monitoring the change in fluorescence of aromatic amino acids at 330 and 350 nm. HSP10 contains 4 tyrosines.


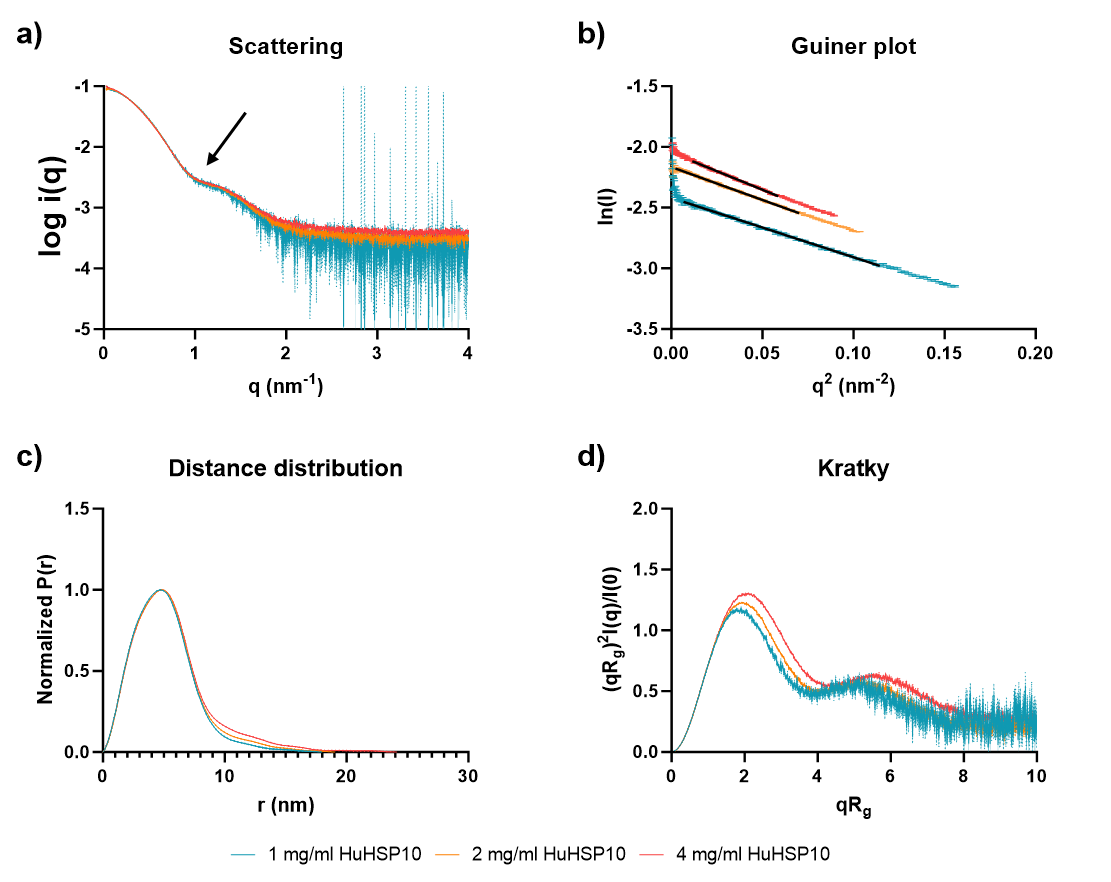


**Figure S2.** SAXS data of HSP10 at different protein concentrations. **a)** Show the scattering of HSP10, Black arrow indicated the dip in the scatter seen in the mid-q region **b)** show the Guinier region picked for the Distance distribution analysis in **c)** and the Kratky plot in **d).**


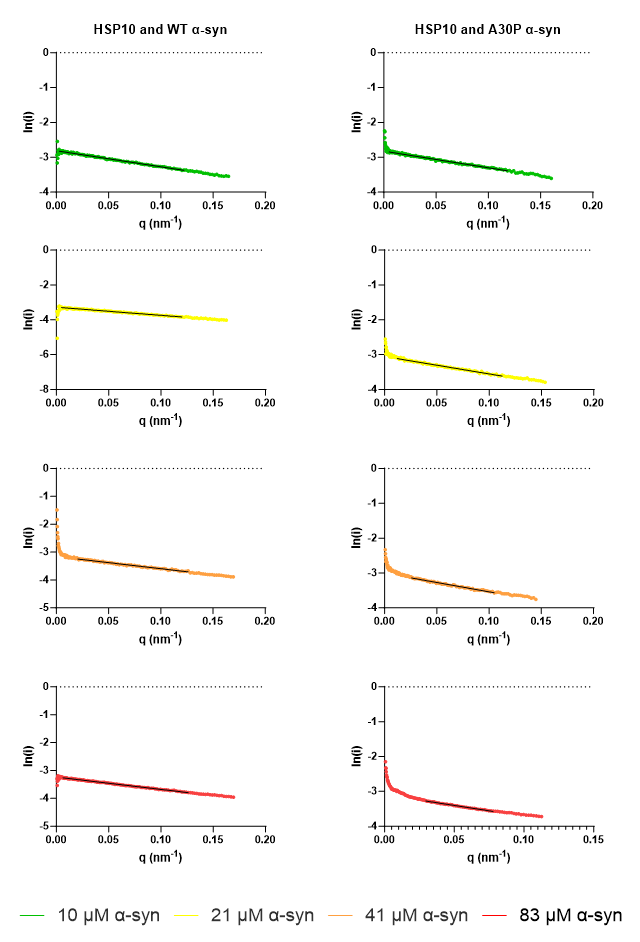


**Figure S3.** Guinier regions for HSP10 with either WT α-syn or A30P α-syn. Black lines show the linear fit.


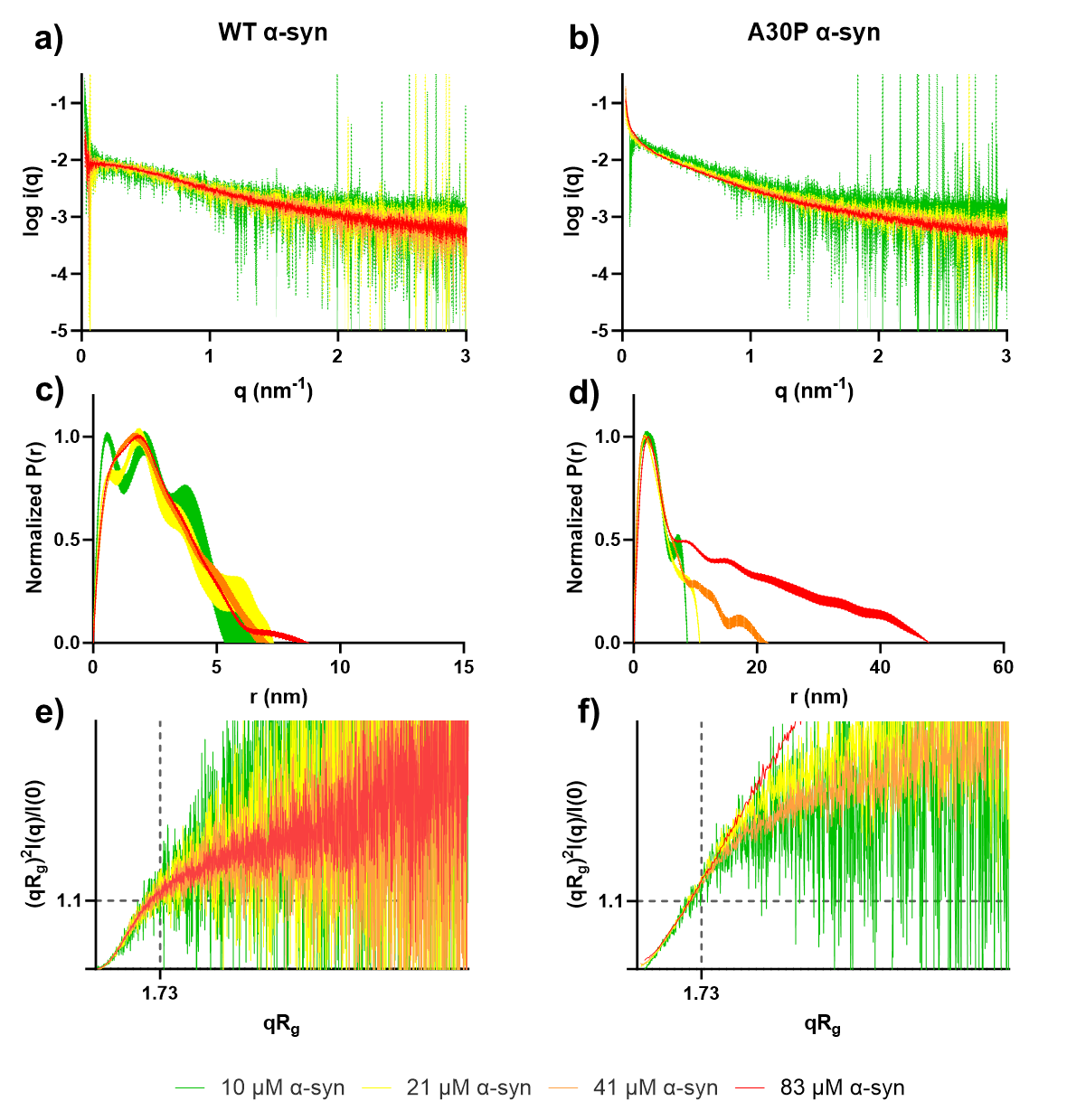


**Figure S4.** SAXS data from different concentrations of α-syn in the absence of HSP10. **a)** and **b)** scattering curves, **c)** and **d)** distance distribution plots and **e)** and **f)** Kratky plots from WT (left) and A30P α-syn (right).

Tables below show the parameters from the SAXS data analysis, following the guidelines form (Trewhella, Jeffries and Whitten, 2023).

**Table S1.** Sample details from SAXS experiments.

**Table S2.** Data collection details from batch measurements.

**Table S3.** Structural parameters of HSP10 with WT α-syn from batch measurements.

**Table S4.** Particle size determination of HSP10 with WT α-syn from batch measurements.

**Table S5.** Structural parameters from form HSP10 with A30P α-syn.

**Table S6.** Particle size determination of HSP10 with A30P α-syn from batch measurements.
